# Development of an Integrated Microfluidic Perfusion Cell Culture System for Real-Time Microscopic Observation of Biological Cells

**DOI:** 10.3390/s110908395

**Published:** 2011-08-29

**Authors:** Lung Lin, Shih-Siou Wang, Min-Hsien Wu, Chih-Chin Oh-Yang

**Affiliations:** 1 Department of Mechanical and Automation Engineering, I-Shou University, Kaohsiung 82445, Taiwan; E-Mails: ljl@isu.edu.tw (J.-L.L.); oohomm@yahoo.com.tw (C.-C.O.Y.); 2 Department of Chemical and Materials Engineering, Chang Gung University, Taoyuan 333, Taiwan; E-Mail: kiddsflish@hotmail.com; 3 Graduate Institute of Biochemical and Biomedical Engineering, Chang Gung University, Taoyuan 333, Taiwan

**Keywords:** microfluidics, cell culture, micropumps, microheaters, ITO glass

## Abstract

This study reports an integrated microfluidic perfusion cell culture system consisting of a microfluidic cell culture chip, and an indium tin oxide (ITO) glass-based microheater chip for micro-scale perfusion cell culture, and its real-time microscopic observation. The system features in maintaining both uniform, and stable chemical or thermal environments, and providing a backflow-free medium pumping, and a precise thermal control functions. In this work, the performance of the medium pumping scheme, and the ITO glass microheater were experimentally evaluated. Results show that the medium delivery mechanism was able to provide pumping rates ranging from 15.4 to 120.0 μL·min^−1^. In addition, numerical simulation and experimental evaluation were conducted to verify that the ITO glass microheater was capable of providing a spatially uniform thermal environment, and precise temperature control with a mild variation of ±0.3 °C. Furthermore, a perfusion cell culture was successfully demonstrated, showing the cultured cells were kept at high cell viability of 95 ± 2%. In the process, the cultured chondrocytes can be clearly visualized microscopically. As a whole, the proposed cell culture system has paved an alternative route to carry out real-time microscopic observation of biological cells in a simple, user-friendly, and low cost manner.

## 1. Introduction

Cell culture has become a basic laboratory operation for various applications, for example, the study of physiology and biochemistry of cells [1,2], or the investigation of the cellular response to environmental stimulations such as drugs [3], or toxins [4]. Cell culture systems are commonly regarded as devices in which cells are cultivated under accurately controlled conditions (e.g., temperature, pH, nutrient, and waste levels). The control of these parameters is crucial for maintaining the consistency of culture conditions, as well as ensuring the survival and proliferation of cells in a manageable manner. In conventional cell culture practices, cells are often cultured in static cell culture containers (e.g., the use of multi-well microplate or Petri dishes as cell culture vessels), and are placed in a cell incubator for providing a stable thermal condition of 37 °C. During the process, the culture medium is normally replaced manually and periodically. In addition, microscopic observation is the commonly-used method to detect the cellular behavior. Under such cell culture format, the cell culture vessels have to be periodically removed from a culture incubator for microscopic examinations.

Although static cell culture systems are simple to operate the culture environment in the conventional static cell cultures may fluctuate due to the intermittent medium replacement processes [5]. In such a poorly controlled environment, the cellular response to the cell culture conditions tested may become more complex because biological cells are very sensitive to the extracellular environment [2]. In some cellular investigations, moreover, a real-time microscopic observation is required. The point-to-point observation in conventional static cell cultures might not meet the demand unless a small scale microscope-compatible incubator is used.

In order to establish a stable culture environment for cellular assays, a perfusion cell culture format is promising because it can continuously provide nutrient supply and waste removal to a cell culture system, and hence keep the culture environment more stable [5,6]. This contributes to a more stable, and thus a more quantifiable extracellular condition, which is found particularly valuable for a precise cellular assay. With the recent progress in microfabrication and microfluidic technology, microfluidic systems have been progressively used as versatile perfusion cell culture tools for various research purposes [3,7,8]. Microfluidic perfusion cell culture systems not only largely reduce the need for experimental resources but also could bring several inherent niche improvements (e.g., providing a more *in vivo*-like culture environment [9]), which have been well reviewed previously [10]. More recently, some microfluidic perfusion cell culture systems [11–14] have been proposed for real-time microscopic observation of cellular images.

In designing a microfluidic perfusion cell culture system for real-time microscopic observation of cellular activities, the mechanisms of culture medium pumping, and thermal control are crucial. The ability to precisely transport and to manipulate a tiny amount of fluid in a microfluidic-based cell culture system is important for medium perfusion, delivery of tested chemicals, or creation of specific microenvironments to the cultured cells. Liquid delivery in microfluidic cell culture systems is commonly achieved by the use of commercially available syringe or peristaltic pumps. However, these lab-scale pumps are bulky, which could thus hamper their integration with the miniaturized cell culture systems. With the rapid development of Micro-Electro-Mechanical Systems (MEMS) and microfluidic technology, micro-scale liquid pumping devices with various mechanical, or non-mechanical actuation mechanisms have been extensively investigated. Among them, the utilization of a pneumatically-actuated, membrane-based micropump, first proposed by Unger and co-workers [15], offers several advantageous features, particularly the simplicity in fabrication and operation, and the ease in integrating into a microfluidic system. Although reports in the literature have demonstrated the feasibility of using pneumatically-driven micropumps for medium pumping in microfluidic perfusion cell culture systems [3,12,16], fluid backflow phenomena may occur in these devices, which could thus cause microbial contamination in the cell cultures.

Moreover, the temperature environment plays a critical role in a cell culture. It has been reported that the thermal environment in a cell culture system could have significant impacts on cell physiology [17,18]. In a general animal cell culture, the temperature (e.g., 37 °C) is normally maintained by the use of commercial cell culture incubators. In an incubator setup, the microscopic observations or other online monitoring activities of cell culture are quite demanding, and thus complicate the experimental operations. Also, traditional cell incubators are commonly bulky and are not readily compatible with the experimental setup for perfusion cell culture, in which interconnections between the medium feeding tubing with the external medium pumping equipment are normally required. These technical hurdles suggest a crucial need for a smart thermal control device, compatible with microfluidic perfusion cell culture operations. With the help of microfabrication technology, various microheaters have been proposed, mainly for micro-scale polymerase chain reaction (PCR) [19], cell lysis [20], or cell culture [21]. Nevertheless, most of the published micro-scale heating devices were fabricated by the technique of evaporation deposition of a metal thin film (e.g., platinum (Pt) or gold (Au)) on a substrate, which is normally complicated and costly to fabricate. Most importantly, the optical transparency of the resulting microheaters is greatly affected by the fabrication process, or the choice of material. This could hinder their application for integrating into a microfluidic perfusion cell culture system for real-time cellular imaging.

To tackle the aforementioned technical hurdles, an integrated microfluidic perfusion cell culture system for real-time microscopic observation of biological cells was proposed. One of the key features of the system is the incorporation of a simple pneumatically-driven micropump coupled with a normally-closed valve for backflow-free medium perfusion in the cell culture chip. Another distinctive feature is the integration of a transparent indium tin oxide (ITO) glass-based microheater chip in the system [22], enabling the creation of stable and uniform thermal conditions in the cell culture chamber. By combining these characteristics, not only does the integrated system provide stable and uniform cell culture conditions, but it also holds great promise for real-time microscopic observation of biological cells. In this study, an integrated system comprising a microfluidic perfusion cell culture chip module, and an ITO-glass microheater chip module was designed, fabricated, and evaluated in terms of its performance. Briefly, the proposed medium pumping mechanism was demonstrated to be able to perform continuous medium perfusion with a flow rate range of 15.4 to 120.0 μL·min^−1^. Numerical simulation and experimental evaluation were also conducted to prove that the ITO-glass microheater chip module was capable of providing a spatially uniform thermal environment in the cell culture chamber, and a precise temperature control with a mild variation of ±0.3 °C. Furthermore, an articular chondrocyte cell culture and a microscopic observation were carried out to demonstrate the feasibility of using the presented cell culture system for micro-scale perfusion cell culture, and its real-time microscopic observation. As a whole, the proposed cell culture system not only features the provision of cell culture conditions with excellent chemical and thermal uniformity, but it also offers an alternative route to carry out real-time microscopic observation of biological cells in a simple and low cost manner.

## 2. Experimental Section

### 2.1. Design

**Figure 1.**
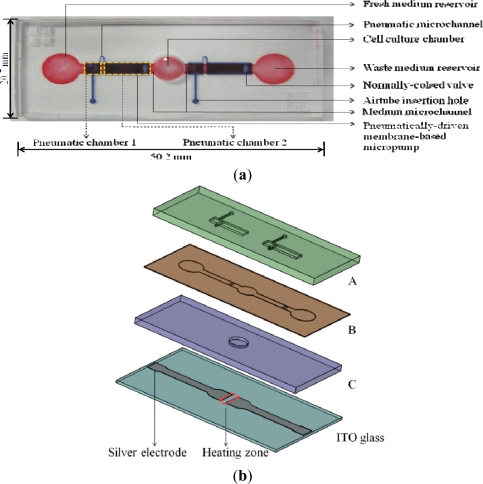
(**a**) Top view layout (photograph) of the microfluidic cell culture chip; and (**b**) The assembly of the integrated microfluidic perfusion cell culture system; A–C: microfabricated PDMS plates, and a silver electrode-patterned ITO glass layer.

The proposed cell culture system integrates the functions of culture medium delivery and thermal control mechanisms for micro-scale perfusion cell culture under a microscope. The system is composed of a perfusion-based microfluidic cell culture chip module, and an ITO glass-based microheater chip module. The layout (photograph) of the microfluidic cell culture chip is shown in Figure 1(a).

It comprises fresh and waste medium reservoirs (D: 5 mm, H: 5 mm), two medium microchannels (W: 2 mm, L: 5 mm, H: 200 μm) located at the both sides of a cell culture chamber (D: 5 mm, H: 200 μm), and two pneumatically-driven membrane-based micropumps located on both sides of the cell culture chamber. In each pneumatic micropump, two pneumatic chambers (pneumatic chamber 1: L: 2.5 mm, W: 2.5 mm, H: 0.5 mm; pneumatic chamber 2: L: 7.5 mm, W: 2.5 mm, H: 0.5 mm) connected by a pneumatic microchannel were designed. In terms of operation, the fresh medium reservoir was designed to be loaded with cell culture medium, and the loaded medium was then driven by the incorporated pneumatically-driven membrane-based micropumps coupled with normally-closed valves [Figure 1(a)]. The assembly of the cell culture chip is schematically illustrated in Figure 1(b). Briefly, three layers of microfabricated PDMS plates (A to C) were permanently bonded by plasma oxidation treatments. This was followed by attaching them to the microfabricated ITO glass, whereby the cell culture chamber is just located on the heating zone of ITO glass. ITO-glass substrate is a transparent conductive material which can generate heat due to its electric resistance when an electric current passes through. Two silver electrodes were patterned on the proposed ITO-glass microheater chip [Figure 1(b)], in which the area (5 × 5 mm^2^) between the silver electrodes serves as a heating zone.

**Figure 2.**
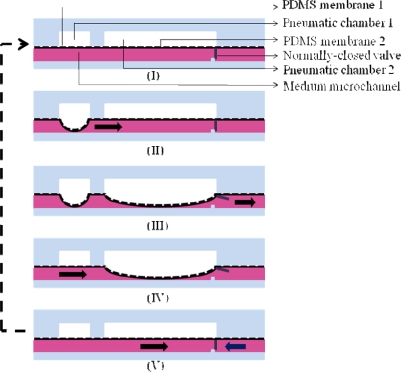
Schematic representation of the medium pumping mechanism using the integrated pneumatically-driven membrane-based micropump coupled with a normally-closed valve (the cross-sectional view) (the arrows indicate the components in the medium pumping mechanism). (**I**) the rest state, (**II**) the deformation of PDMS membrane 1 when its corresponding pneumatic chamber 1 above is pressurized, (**III**) the deformation of PDMS membrane 2 when its corresponding pneumatic chamber 2 is pressurized that also leads to the open of the normally-closed valve, (**IV**) the deformed PDMS membrane 1 regains to its rest state when the pneumatic chamber 1 is depressurized, and (**V**) the deformed PDMS membrane 2 regains to its rest state when the pneumatic chamber 2 is depressurized that also leads to the close of the normally-closed valve.

One of the key features of the proposed cell culture system is the backflow-free medium perfusion function. In this study, two pneumatically-driven membrane-based micropumps coupled with normally-closed valves were integrated to continuously deliver the loaded culture medium flowing from the fresh medium reservoir through the cell culture chamber [Figure 1(a)]. The working principle of medium pumping is based on the pneumatically-driven pulsations of the elastic PDMS membranes located above the medium microchannel to deliver medium forwards. The detailed pumping mechanism is illustrated in Figure 2. Briefly, when the pneumatic chambers of micropumps are pressurized it first causes the deformation of PDMS membrane 1 [Figure 2(II)], and then PDMS membrane 2 [Figure 2(III)]. The sequential effect of the membrane movement is mainly created by the effect of fluidic resistance during the pressurization of the pneumatic chamber 1 and 2 (readers should also refer to Figure 1(a)). In this process, the deformation of the PDMS membrane 1 not only squeezes the fluid in the medium microchannel bidirectionally but, more importantly, functions as an active valve [Figure 2(II)]. The latter ensures that the liquid flow is driven downstream when the PDMS membrane 2 is deformed [Figure 2(III)]. Apart from pumping the liquid forwards, the deformation of the PDMS membrane 2 also mechanically opens the normally-closed valve (an auxiliary elastic PDMS membrane affixed to the PDMS membrane 2; T: 500 μm, W: 2 mm, H: 180 μm), which allows the fluid driven by the deformation of the PDMS membrane 2 to pass through [Figure 2(III)]. Conversely, when the pneumatic pressure in the pneumatic chamber 1 and 2 is released, the deformed PDMS membrane 1 first goes back to its rest state [Figure 2(IV)]. This is followed by the release of membrane 2 [Figure 2(V)], by which the valve again returns to its normally-closed state [Figure 2(V)]. With this approach, any backflow of the pumping fluid can be physically prevented and therefore, the net medium flow is driven downstream.

### 2.2. Microfabrication and Experimental Setup

For the fabrication of the microfluidic cell culture chip, the overall process is based on a CNC machining for PMMA (polymethylmethacrylate) mold making, a PDMS (polydimethylsiloxane) replica molding process, a PDMS spin-coating process, and a plasma oxidation-treated bonding process, which have all been described previously [23]. Briefly, the three PMMA molds for fabricating the PDMS layers A to C with the designed microstructures [Figure 1(b)] were created using a CNC miller (EGX-400, Roland Inc., Japan) equipped with a 0.5 mm drill bit (rotational speed: 26,000 rpm and feed rate: 15 mm·min^−1^). For fabricating the PDMS layers A and C, a PDMS replica molding process was carried out. Briefly, PDMS polymer(Sylgard^®^ 184, Dow Corning, MI, USA) was prepared by thoroughly mixing the PDMS pre-polymer with a curing agent in a ratio of 10:1 by weight. The polymer was then deaerated under vacuum to remove any air bubbles generated during mixing, and this was followed by pouring it onto the two fabricated PMMA molds. After a curing process at 80 °C for 3 h, the cured PDMS layer A and C were then obtained from a de-molding process. For fabricating the PDMS layer B, the prepared PDMS polymer was spun onto the fabricated PMMA mold at a rotational speed of 27,000 rpm, and this was followed by the same curing process as mentioned before. After the PDMS replica molding and spin-coating process, the obtained PDMS plates (A, B and C) were then permanently bonded with the aid of an oxygen plasma treatment. After the assembly process, a borer with a suitable size was used to punch through the PDMS layer A and B at the fresh and waste medium reservoir areas to create two reservoirs. The photograph of the assembled microfluidic cell culture chip is shown in Figure 1(a).

**Figure 3.**
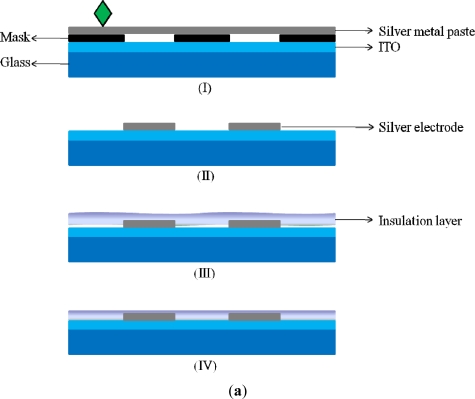

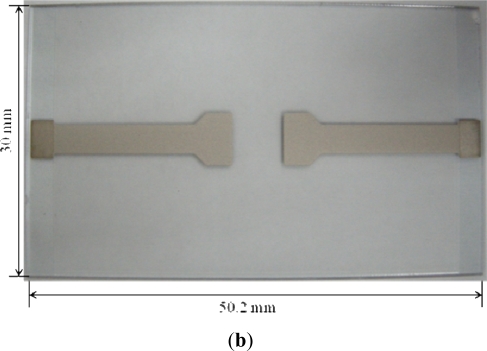
(**a**) Schematic illustration of the fabrication process of ITO-glass microheater chip; (**I**) the mask with a desirable pattern was attached on a ITO-glass substrate and followed by print screening silver metal paste on it, (**II**) after removing the mask, the silver electrodes-patterned ITO-glass substrate was created, (**III**) a 15 μm-thick insulation layer was then screen-printed on the silver electrodes-patterned ITO-glass substrate, and (IV) the fabriacted ITO-glass microheater chip, and (**b**) A photograph of the ITO-glass microheater chip.

For the fabrication of ITO-glass microheater chip, two silver electrodes were directly screen printed on a rectangular ITO glass with a defined heating zone of 5 × 5 mm^2^ (the area in-between the two silver electrodes; Figure 1(b)). The overall fabrication process is schematically illustrated in Figure 3(a). Briefly, a silver metal paste (DW-250H-5, Toyobo Co., Japan) was screen-printed onto an ITO glass substrate (100 Ω/□, Ritek Corp., Taiwan), and was then dried at 150 °C for 40 min [Figure 3(a)-I]. During this stage, the solvent was removed, and the prints (film thickness: 10 μm) adhered onto the ITO-glass [Figure 3(a)-II]. After that, a 15 μm-thick insulation layer (IR-T013PJ, Full-wing Co., Taiwan) was then screen-printed on the top of the fabricated ITO-glass [Figure 3(a)-III], followed by curing at 130 °C for 10 min [Figure 3(a)-IV]. A photograph of the fabricated ITO-glass microheater chip is shown in Figure 3(b).

In order to command the integrated micropumps pneumatically, and the proposed ITO-glass microheater thermally, a custom-made control device was used. For the pneumatic control, two air tubes were utilized to connect the microfluidic cell culture chip [via the holes for air tube insertion; Figure 1(a)], and the control device. In the control device, an air compressor (MDR2-1A/11, Jun-Air Inc., Japan), two electromagnetic valves (EMV, S070M-5BG-32, SMC Inc., Taiwan), and a programmable control circuit system were integrated to activate and control the pneumatic micropumps, based on our previous work [3,16]. For the thermal control, a micro-controller (MPC82G516, Megawin Tech. Co., Taiwan) with a 10-bit analog-to-digital converter (ADC) and an 8-bit pulse-width-modulator (PWM) was used [22]. The thermal control mechanism is based on a general feedback control loop. Briefly, the thermocouple wire, inserted into the cell culture chamber, served as a sensor to monitor the local temperature conditions of the target area where the thermal field needs to be closely regulated. Then, the generated signal was input to the micro-controller module, whereby the output electric current and thus the corresponding temperature conditions on the ITO-glass microheater chip were constantly modulated.

### 2.3. Evaluation of Medium Pumping Rate

In order to evaluate the pumping performance of the integrated pneumatically-driven micropumps coupled with normally-closed valves, the volumetric pumping rates at various driving frequencies (5 to 30 Hz), and magnitudes (5, 10 and 15 psi) of the applied pneumatic pressure were measured. The evaluation was carried out by measuring the weight of liquid output within a period of 4 hours using an electronic balance (AB54-S, Mettler Toledo, Taiwan; readability: 0.1 mg, repeatability: 0.1 mg) [3,16]. The measured weight of liquid output was then converted to a volume of liquid assuming a constant density of water (1 g·cm^−3^). Three separate experiments were conducted to obtain the flow rate data.

### 2.4. Evaluation of Thermal Uniformity

In order to investigate the temperature field distribution inside the cell culture chamber, a numerical simulation was carried out. Since the medium flow rate in the cell culture chamber was low, it is reasonable to assume that the heat transfer was dominated by heat conduction. In this study, therefore, heat radiation was neglected and only heat conduction within the liquid sample, the solid PDMS chamber wall, microchannels, and the ITO glass substrate were considered [22]. A 3-dimensional (3-D) heat conduction model was used to describe the problem. To verify the measured steady-state temperature contours, a 3-D conduction simulation was performed using the CFD software (CFD-ACU+, CFD-RC, USA). Natural free convection condition was imposed on the top and surrounding surfaces of the microfluidic cell culture chip. A constant heat generation rate was assumed to apply on the ITO-glass microheater chip. In this study, the numerical geometry was mainly constructed by four parts: (1) the cover of cell culture chamber [in PDMS layer A, Figure 1(b)]; (2) cell culture chamber and its wall [in PDMS layer B and C, Figure 1(b)]; (3) the bottom of cell culture chamber [in PDMS layer C, Figure 1(b)]; and (4) the two medium microchannels. The numerical dimension of the microfluidic cell culture chip is 10 × 10 × 1.2 mm^3^. In this work, a 3-D numerical domain was discretized into approximately 600,000 cells with structured hexahedral meshes. In addition, the non-uniform mesh grids were used for these numerical simulations. Dense grids were used in the wall regions of the cell culture chamber. A residuals criterion (less than 10^−6^) was used to guarantee the convergence of the solution. Some critical parameters required for the simulation are listed in Table 1.

Apart from the numerical simulation, a thermal infrared (IR) imager (Infrared Thermography TVS-200N, Nippon Avionics Co Ltd., Japan) was used to explore the uniformity of thermal field on the proposed cell culture system. Before the observation, the accuracy of the temperature measurement was checked to be ±1 °C. In this work, the focal plane was perpendicular to the detective light, and the measurement distance to the focal target was set to be 30 cm. In this evaluation, the focal target was the top surface of microfluidic cell culture chip (the emissivity for the IR imaging was set as 0.96 for the PDMS material).

### 2.5. Demonstration for Perfusion Articular Chondrocyte Culture and Microscopic Observation

Articular chondrocyte cell culture under a microscope was performed to demonstrate the feasibility of using the proposed system for perfusion cell culture, and the real-time microscopic observation of the cultured cells. Before loading with cell suspension, the bottom surface of the cell culture chamber was treated with 0.01% fibronectin solution (unless otherwise stated all chemicals were obtained from Sigma, Taiwan) for 1 hour to enhance chondrocyte attachment. After that, 50 μL of cell suspension (10^5^ cells mL^−1^) was loaded into the fresh medium reservoir, and was then delivered to the cell culture chamber using the incorporated pneumatic micropumps. This was followed by incubating under a static state for 1 day to allow cell attachment. After the cell seeding process, perfusion chondrocyte culture was carried out for up to 3 days. In the process, the microfluidic cell culture chip with the ITO-glass microheater chip attached was placed on a dark field microscope (Olympus, BX51) for real-time microscopic observation. In this research, the Dulbecco’s Modified Eagle’s Medium (DMEM) (with 1,000 mg·L^−1^ glucose, 25 mM HEPES, without sodium bicarbonate), supplemented with 10% fetal bovine serum (Invitrogen, Taiwan), 2% antibiotic/antimycotic solution, and 50 μg·mL^−1^ ascorbic acid, was continuously perfused to the cell culture chamber at the set flow condition (flow rate: 1.5 mL·h^−1^, running time: 2 s, frequency: 4 runs·h^−1^). After cell culture, the viability of the articular chondrocytes was evaluated, based on the published works [3,16]. Briefly, a fluorescent dye kit (LIVE/DEAD^®^ Viability/Cytotoxicity Kit L-3224, Molecular Probes), and a fluorescence microscope were used to stain, and to detect the cultured cells, respectively. A digital camera was used capture the images. Cell viability was then quantified by counting the live (green) and dead (red) cells using a software program (SimplePCI version 5.2.1, Compix Inc.).

## 3. Results and Discussion

### 3.1. Performances of Pneumatically-Driven Micropumps Coupled with Normally-Closed Valves for Backflow-Free Medium Perfusion

In this study, a perfusion-based, microfluidic cell culture format was utilized to create a stable and well-defined culture condition for precise cellular assays [24]. In order to carry out medium pumping in a microfluidic system, the utilization of a pneumatically-driven membrane-based micropump holds great promise. The working principle of such liquid pumping scheme is based on the pulsation movements of elastic membranes pneumatically driven by their corresponding pneumatic chambers, which generate a continuous peristaltic effect for pumping a fluid forward [15]. Borrowing from the concept, pneumatic micropumps with various designs have been successfully demonstrated for delivering liquid in microfluidic systems [3,16,21,23,25]. Among them, pneumatic micropump designs with spiderweb-like [3,23], and serpentine-shaped [16,21,25] layouts are the typical configurations. These pneumatic micropumps commonly involve the use of multiple elastic membranes actuated by their corresponding pneumatic chambers to drive fluids. In these designs, the precise control of membrane movements in the micropumps is critical to achieve an optimum pumping performance [25]. However, this is, to some extent, technically demanding. Most importantly, unwanted fluid backflow could occur in these micropumps, which might hamper the precise manipulation of fluid flow in a microfluidic system. For microfluidic cell culture systems, the backflow of pumping liquid might lead to the cross contamination between solutions, or microbial contamination in the cell culture chambers.

For achieving backflow-free liquid pumping some earlier works [26,27] have incorporated valving mechanisms in the pneumatic micropump designs. In the design a liquid flow microchannel, three underlying pneumatic chambers, a flexible PDMS membrane sandwiched in between, and a PDMS floating block structure are configured. The floating block serves as a normally-closed valve to physically prevent the backflow of the pumping fluid. In these cases, a higher flow rate is normally required to produce a relatively higher hydrodynamic pressure in order to open the valve located downstream, by which the pumping fluid can pass through. Although backflow-free liquid pumping can be realized by the design the pumping rate range of these micropumps is relatively high. This could limit its application for micro-scale perfusion cell culture because it might generate a high fluid shear environment, which could damage the living cells. Moreover, the structure, and thus the fabrication process of these pneumatic micropumps are complex. For achieving backflow-free liquid pumping without too much technical complexity, the design of a simple pneumatic micropump coupled with a normally-closed valve has been adopted in this study. The working principle was described in detail in Figure 2. Different from the previous designs [26,27], only one elastic PDMS membrane and its corresponding pneumatic chamber are required to achieve liquid pumping, largely simplifying the fabrication and operation process. In the presented work, moreover, an elastic PDMS partition connected to the pulsating PDMS membrane (Figure 2) functions as an active valve to prevent liquid backflow. In contrast with the earlier works [26,27], such design was capable of well coordinating the movements of both liquid pumping and valving, making the control of liquid delivery in a microfluidic system easier.

**Figure 4.**
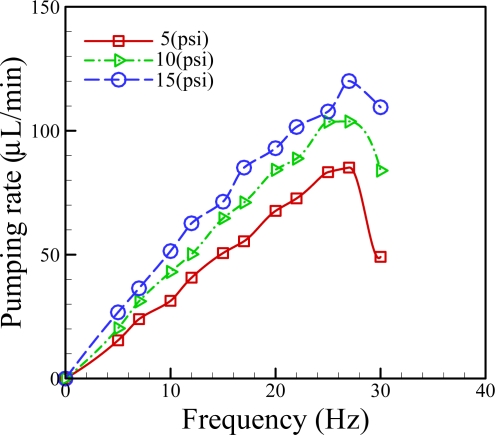
Liquid pumping rate profiles of the integrated pneumatic micropump at various applied pneumatic pressures (5, 10, and 15 psi) and frequencies (5–30 Hz).

In the proposed medium pumping scheme, the volumetric flow rate is dominated by the applied pneumatic pressure, and its frequency. To find out the quantitative links between them, the volumetric pumping rate measurements at various applied pneumatic pressures (5, 10 and 15 psi) and frequencies (5–30 Hz) were carried out. Results (Figure 4) revealed that, within the experimental conditions investigated, the pumping rate profiles showed the similar pattern, in which the pumping rate increased first with the increase of applied frequency, and followed by a decline with the saturation flow rate occurred at the frequency of 26 Hz.

The decline of liquid pumping rate observed in the investigations was mainly caused by the lagging mechanical response of PDMS membranes to high frequencies. This phenomenon was also observed in previous publications [3,16]. In addition, it can be observed from the Figure 4 that the liquid pumping rates increased with the increase of the pneumatic pressure applied. This phenomenon can be reasonably explained by the fact that the higher applied pneumatic pressure can accordingly cause a higher degree of membrane deformation, whereby more liquid in the microchannel was squeezed forwards. As a whole, the proposed medium pumping mechanism was demonstrated to be able to perform medium perfusion with a flow rate range of 15.4 to 120.0 μL·min^−1^.

### 3.2. Stability and Uniformity of Thermal Condition for Cell Culture

**Figure 5.**
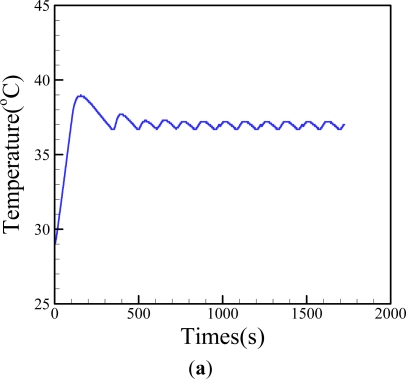

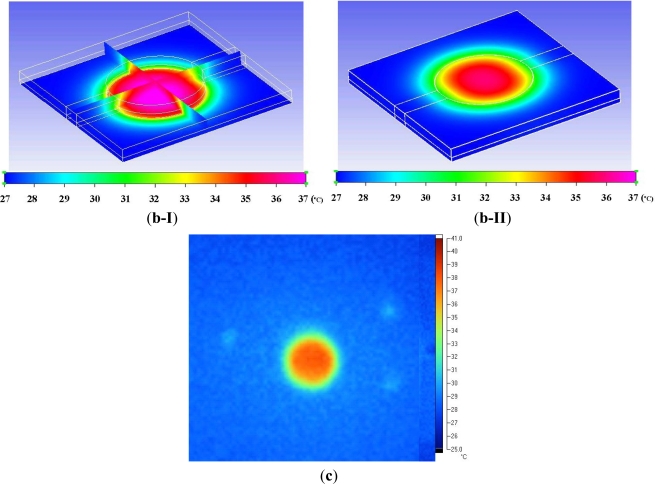
(**a**) Observation on the temperature profile over time (the set temperature was 37 °C and the temperature variation was evaluated to be within ±0.3 °C); (**b**) Numerical simulation-based evaluation of the temperature distributions in the (I) cell culture chamber and (II) on the microfluidic cell culture chip; and (**c**) 2-dimensional thermal IR images on the microfluidic cell chip at the set temperature of 37 °C (top-side view; the circular area represents the cell culture chamber area).

Reports in the literature have demonstrated that the thermal environment in a cell culture system could have significant impacts on cell physiology [17,18]. For a precise cell culture-based assay, the control of temperature conditions is vital. Different from the conventional way of using commercial cell incubators, this study intended to simply use a fabricated ITO-glass, and its associated thermal control system, to carry out the same task, based on our previous experience [22]. This is mainly due to its low cost, mobility, simplicity, and transparency. The features facilitate the real-time microscopic observation of the cultured cells. In this study, the temperature generated by the proposed ITO-glass microheater chip was closely regulated by a feedback control loop through a control device as aforementioned. To ensure the temperature conditions maintained by the presented system were stable, experimental validation was carried out. Figure 5(a) shows that the proposed thermal system was capable of providing a steady temperature field with a reasonable deviation of within ±0.3 °C, which is suitable for a general cell culture practice.

To examine the homogeneity of the thermal field in the cell culture chamber, moreover, numerical simulation was carried out. To mimic the real cell culture conditions, the cell culture chamber and the two medium microchannels were assumed to be filled with water. The heat transfer phenomenon occurred in the proposed system, PDMS-based microfluidic cell culture chip with an ITO-glass microheater chip attached, is mainly governed by the mechanisms of thermal conduction inside, and free convection outside the system. Free convection effects are difficult to determine due to their complexity. Convection heat transfer coefficient (h) is the function of surface geometry, the nature of fluid motion, the properties of the fluids, and the bulk fluid velocity. In this simulation, the value of h was set as 7.5 Wm^−2^·K^−1^ [28] to evaluate the convective heat transfer in the case of ITO power generation (*q* = 1.5 × 10^5^ W·m^−3^) for approximation [22].

The simulation results [Figure 5(b)] revealed that the thermal distribution was spatially uniform (37 ± 1 °C) in the central area of cell culture chamber [Figure 5(b)-I], and was homogeneous on the central surface of cell culture chamber [Figure 5(b)-II], indicating the proposed thermal control scheme was capable of generating a uniform thermal environment for cell culture. To justify the previous thermal simulation, experimental evaluation was carried out using a thermal IR imager. In this evaluation, the microfluidic cell culture chip was attached onto the ITO-glass microheater chip, and followed by filling cell culture chamber with cell culture medium to mimic the real cell culture setting. Figure 5(c) shows the thermal IR image on the surface of the microfluidic cell culture chip at the set temperature of 37 °C. It was clearly observed that the temperature field on the central cell culture chamber (the orange color area) was uniformly kept at the set temperature. In this measurement, a ring of light green (33 °C) around the cell culture chamber was observed. This observation is consistent with the numerical simulations [Figure 5(b)-II]. This is mainly due to the fact that the thermal conductivity of PDMS material is lower than that of water. Notably, moreover, both of the simulated and measured temperature profiles show a round temperature feature. This phenomenon can also be explained by y the fact that the conduction coefficient of the liquid-filled cell culture chamber is much higher than that of the cylindrical chamber walls (PDMS material). Therefore, heat flux generated by the ITO glass heater is mainly transferred through the liquid medium. As a whole, the results above have demonstrated the feasibility of using the fabricated ITO-glass microheater chip and its associated control system to provide a stable and uniform thermal field for cell culture.

### 3.3. Demonstration for Perfusion Articular Chondrocyte Culture and Microscopic Observation

In order to demonstrate the feasibility of using the integrated microfluidic perfusion cell culture system for a cell culture practice, and its real-time microscopic observation, articular chondrocyte cell culture was performed. In the study, the incorporated pneumatic micropumps were used to first deliver the fibronectin solution for surface treatment, the cell suspension for cell seeding, and finally the culture medium for a 3-day cell culture.

**Figure 6.**
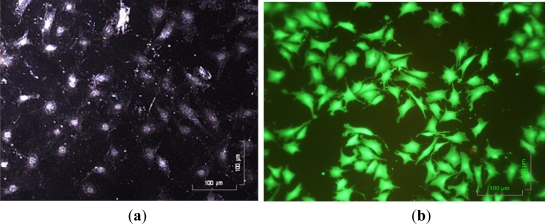
(**a**) The observation of articular chondrocyte morphology during cell culture period using a dark field microscope; and (**b**) the observation of cell viability after 3 day perfusion cell culture using the Live/Dead^®^ fluorescent dye and fluorescent microscope (Green and red dots represent live and dead cells, respectively).

In the operations, these solutions were loaded into the fresh medium reservoir in order, and were sequentially delivered through the integrated micropumps. Due to the normally-closed valve design, no fluid backflow was observed, largely minimizing the risk of cross contamination between solutions. This solves the technical problems commonly observed in the previous pneumatic micropump designs [3,16,21,23,25]. During cell culture, the cultured cells can be observed microscopically in a real-time manner. Figure 6(a) shows that the cultured chondrocytes can be clearly visualized. After 3 day perfusion cell culture, moreover, the cell viability was observed, and estimated using a fluorescent dye kit and microscopic observation. It can be clearly seen from Figure 6(b) that the cell viability of the cultured cells was as high as 95 ± 2%, indicating that the proposed system was capable of performing a long-term perfusion cell culture at a micro scale. As a whole, this study has developed a simple, and user-friendly micro-scale cell culture platform that is particularly suitable for real-time microscopic observation of cell culture.

## 4. Conclusions

In this study, a microfluidic perfusion cell culture system was developed for micro-scale cell culture practices, and for their online monitoring (e.g., microscopic observation). Due to the miniaturized cell culture scale and perfusion cell culture format, the proposed cell culture system was able to create a more stable, well-defined, and thus more quantifiable culture condition, enabling researchers to precisely investigate the quantitative links between the cellular responses, and the tested culture conditions. In order to achieve this, the mechanisms of culture medium pumping, and thermal control were incorporated in the cell culture system, largely eliminating the need for a commercial liquid pumping equipment, or cell incubator. This makes the setup for perfusion cell culture more compact, and thus facilitates the real-time monitoring activities of cell culture. In this work, the integrated microfluidic perfusion cell culture system consisting of a microfluidic cell culture chip, and an indium tin oxide (ITO) glass-based microheater chip was designed, fabricated, and experimentally evaluated in terms of its performance. Results showed that the pneumatically-driven, membrane-based micropumps coupled with normally-closed valves were able to provide liquid pumping rates ranging from 15.4 to 120.0 μL·min^−1^. In the operation, no fluid backflow was observed, thus largely minimizing the cross contamination between solutions and microbial contamination in the cell culture chamber. Also, the proposed micropump design greatly simplified the structure, fabrication and operation process, in contrast with the published works. Moreover, the presented ITO-glass microheater chip was proved to be capable of providing a spatially uniform thermal environment, and precise temperature control with a mild variation of ±0.3 °C, which is suitable for a general cell culture practice. The use of transparent ITO-glass as a microheater not only benefits the microscopic detection works, but also makes the experimental setup simple, portable, and thus facilitates the real-time monitoring of cell cultures. Different from the conventional approaches to fabricate an ITO-glass-based microheater, this study simply used a print screen process to construct silver electrodes on an ITO glass, making the fabrication work cost-effective, and simple. Furthermore, an articular chondrocyte perfusion cell culture was successfully demonstrated using this proposed cell culture system, showing the cultured cells were kept at high cell viability of 95 ± 2%. In the process, the cultured chondrocytes can be clearly visualized microscopically. As a whole, the proposed cell culture system has paved an alternative route to carry out real-time microscopic observation of biological cells in a simple, user-friendly, and low cost manner.

## Figures and Tables

**Table 1.** Material properties of glass, PDMS and water under the conditions of temperature: 298 K and pressure: 1 atm.

**Material**	**Conductivity: κ (W/mK)**	**Density: ρ (kg/m^3^)**	**Specific heat: C (J/kgK)**
Glass	1.4	2,230	840
PDMS	0.15	970	1,460
Water	0.613	997	4,160
